# Gene co-expression network analysis reveals immune cell infiltration as a favorable prognostic marker in non-uterine leiomyosarcoma

**DOI:** 10.1038/s41598-021-81952-8

**Published:** 2021-01-27

**Authors:** Mohammad Darzi, Saeid Gorgin, Keivan Majidzadeh-A, Rezvan Esmaeili

**Affiliations:** 1 Department of Electrical Engineering and Information Technology, Iranian Research Organization for Science and Technology (IROST), Tehran, Iran; 2 Genetics Department, Breast Cancer Research Center, Motamed Cancer Institute, ACECR, Tehran, Iran

**Keywords:** Cancer, Systems biology

## Abstract

The present study aimed to improve the understanding of non-uterine leiomyosarcoma (NULMS) prognostic genes through system biology approaches. This cancer is heterogeneous and rare. Moreover, gene interaction networks have not been reported in NULMS yet. The datasets were obtained from the public gene expression databases. Seven co-expression modules were identified from 5000 most connected genes; using weighted gene co-expression network analysis. Using Cox regression, the modules showed favorable (HR = 0.6, 95% CI = 0.4–0.89, *P* = 0.0125), (HR = 0.65, 95% CI = 0.44–0.98, *P* = 0.04) and poor (HR = 1.55, 95% CI = 1.06–2.27, *P* = 0.025) prognosis to the overall survival (OS) (time = 3740 days). The first one was significant in multivariate HR estimates (HR = 0.4, 95% CI = 0.28–0.69, *P* = 0.0004). Enriched genes through the Database for Annotation, Visualization, and Integrated Discovery (DAVID) revealed significant immune-related pathways; suggesting immune cell infiltration as a favorable prognostic factor. The most significant protective genes were ICAM3, NCR3, KLRB1, and IL18RAP, which were in one of the significant modules. Moreover, genes related to angiogenesis, cell–cell adhesion, protein glycosylation, and protein transport such as PYCR1, SRM, and MDFI negatively affected the OS and were found in the other related module. In conclusion, our analysis suggests that NULMS might be a good candidate for immunotherapy. Moreover, the genes found in this study might be potential candidates for targeted therapy.

## Introduction

Sarcomas are heterogeneous and rare mesenchymal malignancies that are originated from different tissues. The sarcomas' biological characteristics are not understood well due to the high heterogeneity and uncommonness of this disease. Leiomyosarcoma (LMS), which are originated from smooth muscle cells, accounts for 14% of sarcomas and are the most popular soft tissue sarcomas^1^. Microarray analysis divides the LMSs into three subtypes. Subtype I expresses muscle associated genes, subtype II shows no significant differentiation from smooth muscle, and subtype III shows specific anatomic sites and is originated from the uterus^2^. In recent years, the efforts to explain the molecular heterogeneity of LMS have been increased. High throughput technologies generate opportunities to create new insight into different aspects of biological systems. This opportunity may compensate for the rare number of clinical trials in finding new LMS treatments in the future.

There are some studies on gene expression analysis of LMS^3–5^. Some discovered genes were differentially expressed in LMS in comparison with healthy tissues^3^. Moreover, higher expression of BCL2-associated agonist of cell death (BAD), SRC proto-oncogene, non-receptor tyrosine kinase (SRC), serum response factor (SRF), and myocardin (MYOCD) were confirmed in LMS in comparison with other subtypes of sarcomas^6^. Loss of fragments in chromosomes 1, 4, 16, and 18 were also reported in comparative genome hybridizations in LMS^7,8^. Despite many distinguishing efforts to find treatment options by identifying gene expression levels in LMS, surgery is still the main treatment. The currently available systematic therapies are not always effective in this cancer. Moreover, no targeted therapy exists, and personalized medicine approaches seem far away in LMS management. This situation is exacerbated in metastatic LMS. In other cancers, estimating the prognosis of the patients help to decide about the appropriate treatment^9,10^. But, the studies reporting the effect of gene expression in the survival of patients with LMS are rare^11^.

Most of the investigations on LMS gene expression has used differential expressed genes (DEGs). Although DEGs elicit vital information from high throughput data, it has some limitations. In fact, in DEG analyses, individual genes are identified, so the interactions between genes are ignored. In other words, DEGs fail to recognize the expression and organization of thousands of genes simultaneously. Gene expression is highly regulated, and it forms a pattern of co-expression networks in cells^12^. It is hypothesized that most of the time, carcinogenesis is not the result of several genes' deregulation. It is the consequence of complex mechanisms, such as subtle interconnection between genes in the regulatory networks^13^. Learning such patterns is crucial in cancer-associated studies that cannot be obtained with simple DEGs. To the best of our knowledge, no research has focused on non-uterine leiomyosarcoma (NULMS) based on gene interaction networks in recent years. However, a study was published that investigated all types of LMSs together^14^.

Weighted gene co-expression analysis (WGCNA) is a general framework that provides a system biology approach. By applying WGCNA, detailed characteristics have been investigated at the genetic network level^15^. This framework has been successfully utilized to study different cancers and non-cancer diseases^16,17^.Finding co-expression patterns can also associate the unknown function genes with biological processes due to the guilt-by-association (GBA) basis of WGCNA.

In this paper, the authors utilized the WGCNA algorithm as a system biology method to identify critical co-expressed genes and hub genes; affecting the NULMS survival. Eventually, the function, cellular compartment, and pathways related to patients' relapse were investigated through gene ontology. The study aimed to improve the understanding of NULMS prognostic genes through constructing a co-expression network with RNA sequencing data.

## Results

### Network construction reveals seven co-expression modules

We were interested in identifying clusters (modules) of co-expressed genes from transcriptomic data of NULMS. A network module is a subset of nodes that forms a sub-network inside a larger network. Soft and hard thresholding are two approaches to construct a co-expression network. WGCNA is a framework principally proposed for analyzing weighted networks. In this study, the soft-thresholding approach was selected to build the NULMS co-expression network.

**Figure 1:**
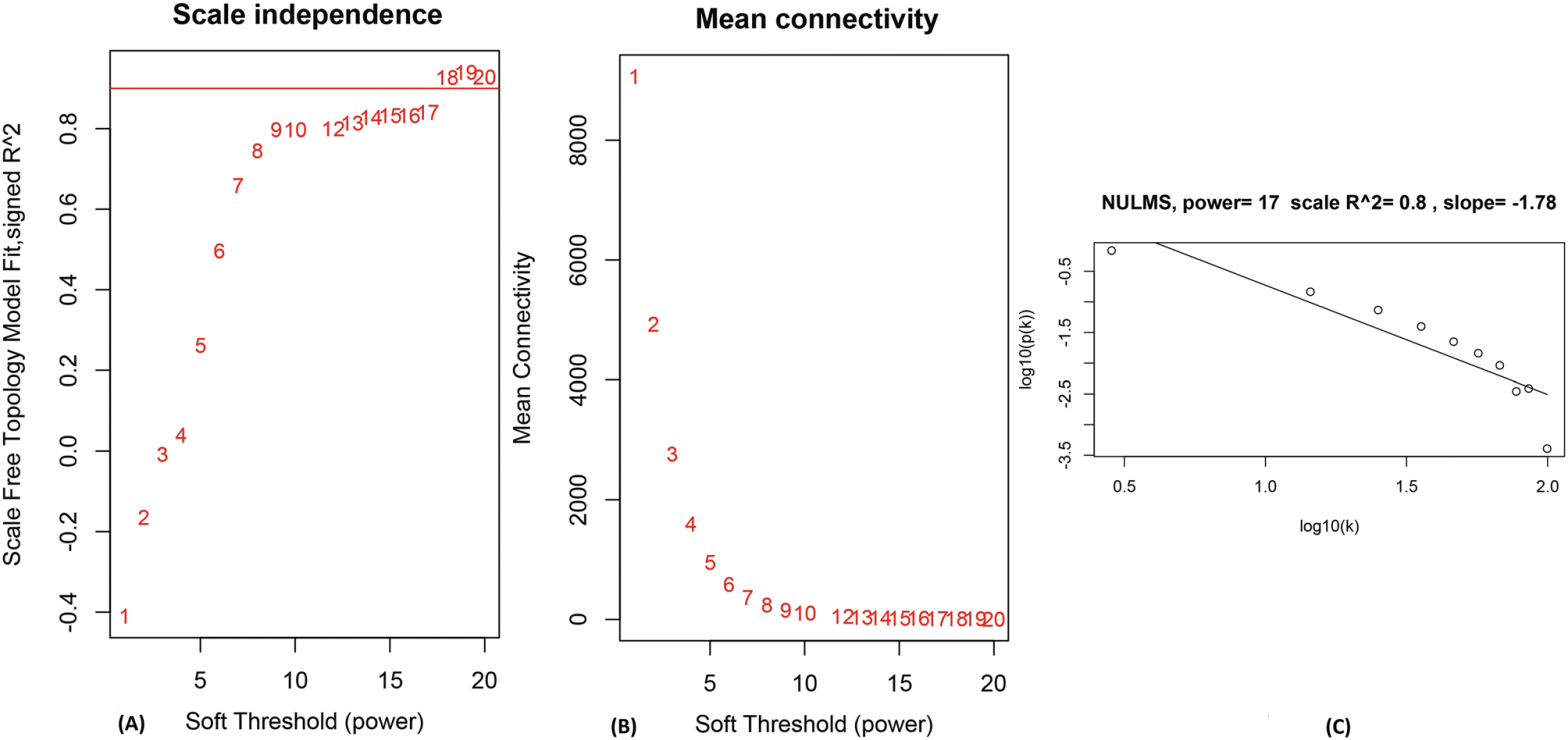
Analysis of network topology for several soft-thresholding powers in WGCNA. (**a**) Scale-free fit index for different powers (β). (**b**) Mean connectivity analysis for various soft-thresholding powers (β). (**c**) Linear model fitting of R2 index showed quality of relationship between connectivity (k) and P(K).

The parameter β is essential for fulfilling the scale-free topology property of the co-expression network. Biological networks which are based on gene expression data are most likely to be scale-free^18^. Therefore, β = 17 was considered to obtain scale-free topology by the fit index greater than 0.8. Figure 1 shows the result of several powers for finding a network with scale-free topology properties.

**Figure 2:**
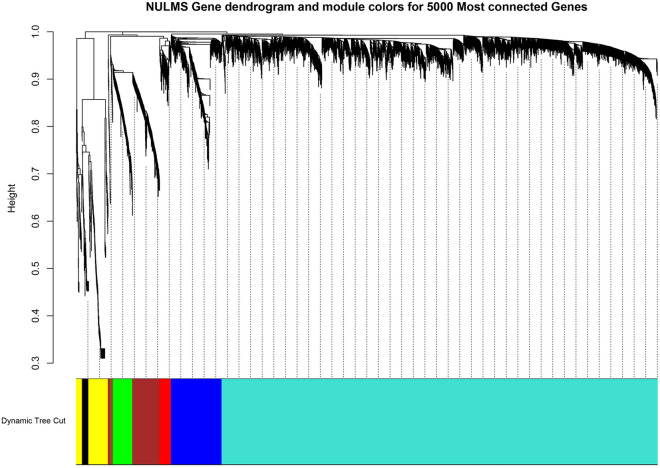
Gene dendrogram and module colors for TCGA NULMS.

The adjacency matrix was then produced through the adjacency function; using the β and gene expression matrix. The hierarchical clustering was built based on the TOM dissimilarity measure, as shown in Fig. 2. We identified seven co-expression modules. From large to small, these modules are turquoise, blue, brown, yellow, green, red, and black, respectively. In this study, each gene was assigned to separate modules.

### Module validation

**Figure 3:**
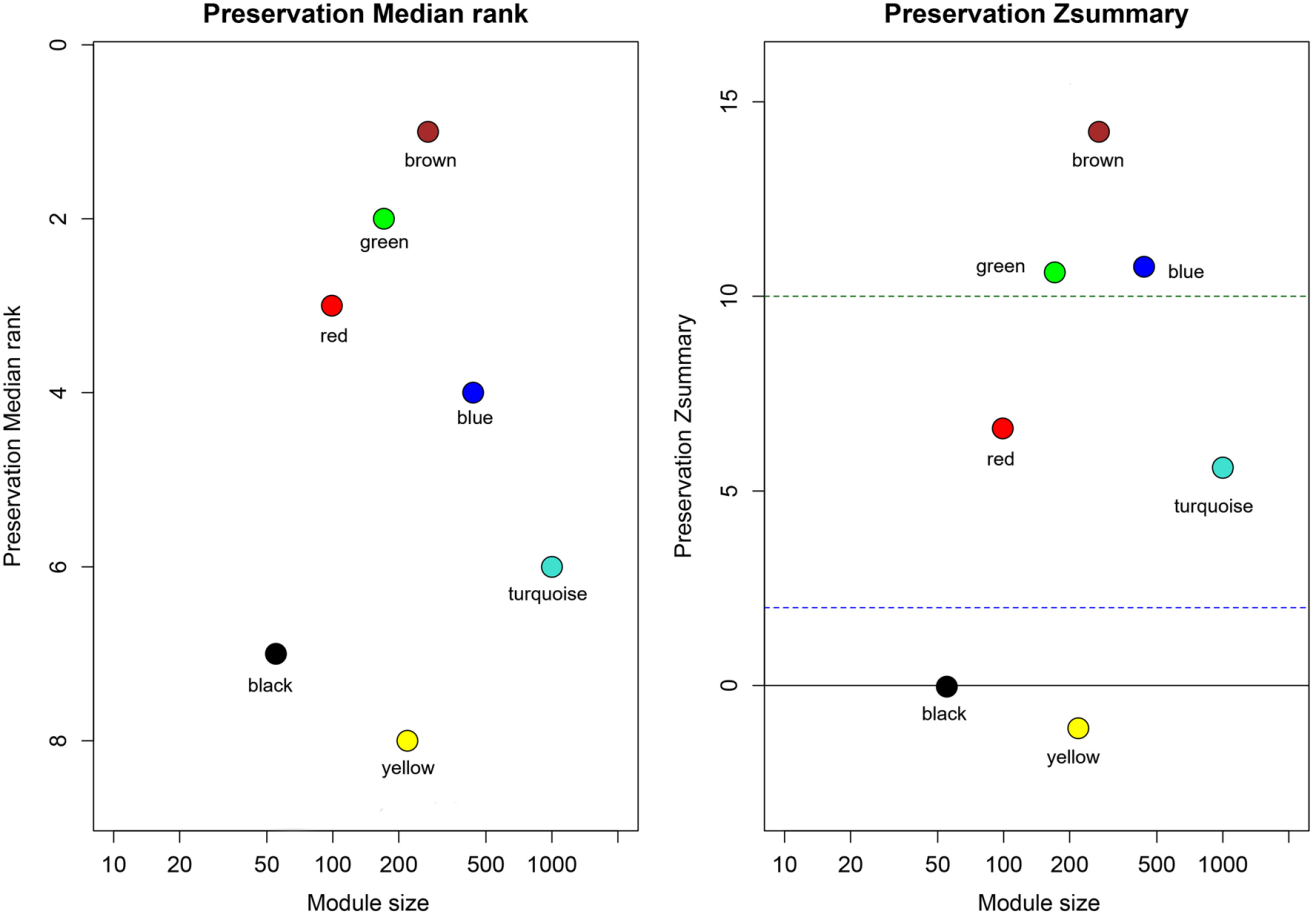
Median rank and Z-summary statistics in the module preservation process. (**a**) The plot shows the module position in the test dataset based on the Median rank. (**b**) The plot illustrates the analysis of the Z-summary between different modules in the test dataset.

Identified co-expressed modules in the reference dataset should be examined for validity with a standalone dataset. We used the module preservation statistics to achieve reliable and preserved modules. To this end, the co-expression network was constructed again by the NULMS Stanford dataset, and genes were assigned to modules based on the module assignment scheme in the reference dataset. Figure 3 shows that blue, brown, and green modules are strongly preserved (i.e., Z-summary more than 10); while the red and turquoise are moderately preserved (i.e., 5 < Z-summary < 10). The median rank of the green and black module is 2 and 7, respectively. Those values indicate that the green is more strongly preserved than the black module.

### Prognostic modules identification

#### Module–trait relationship

**Figure 4:**
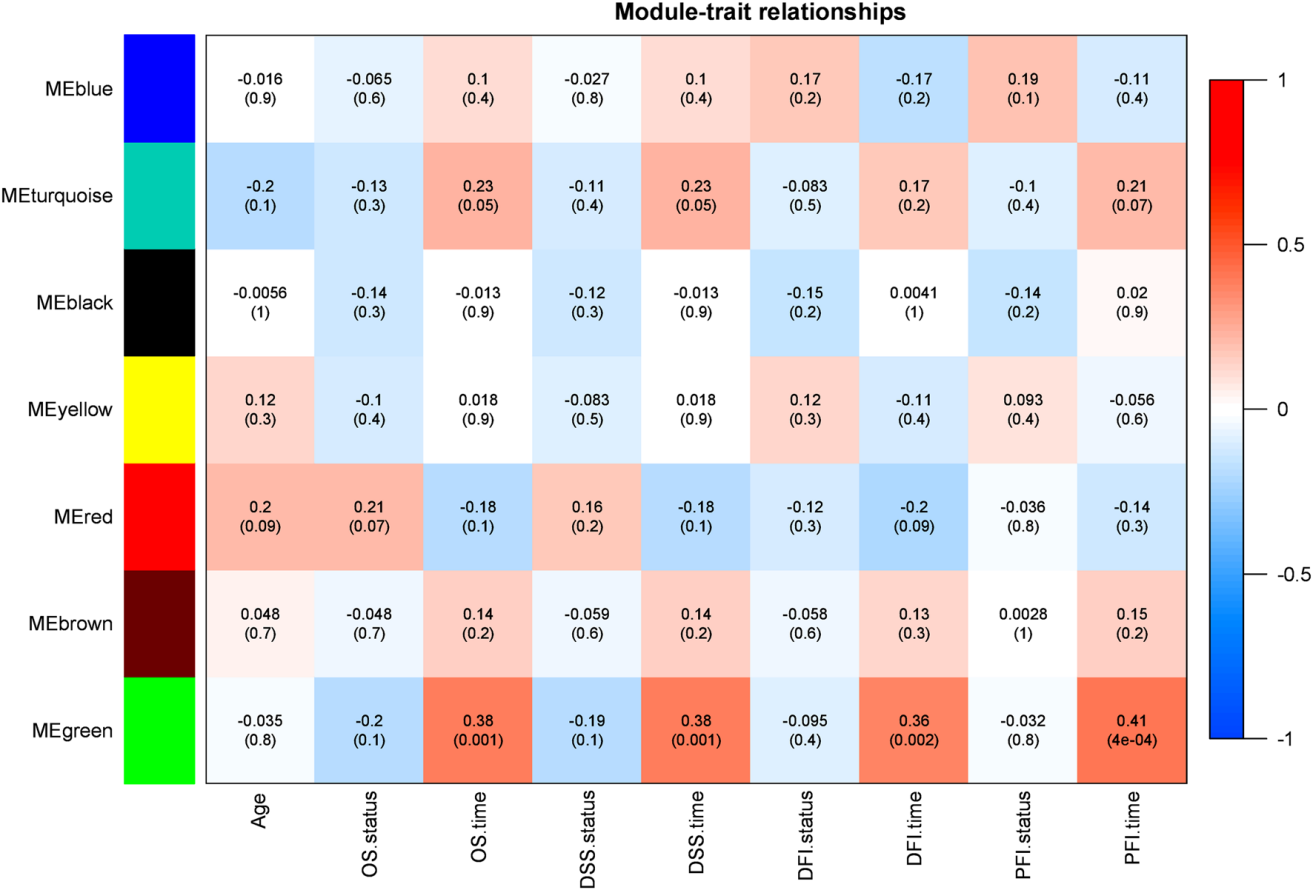
The module–trait relationships were demonstrated by correlation values and *P* values (In parenthesis) with a range of colors; the degree of correlation between modules and clinical features is shown. Rows are module eigengene (ME) regards to each module, and the columns indicate traits.

Finding the relationship between gene expression profiles and clinical traits is one of the WGCNA framework's advantages. The association between module eigengenes and clinical information such as age, different survival status, and time was computed through the Pearson’s correlation coefficient. Moreover, the *P* value was calculated for the given correlation. As shown in Fig. 4, the green module had a significant correlation with survival endpoint times including overall survival (OS), disease-specific survival (DSS), and progression-free interval (PFI) (*P* < 0.05).

#### Survival analysis

In this study, we were interested in finding the effect of significant modules on patients’ survival. For this purpose, we used module eigengene (ME) as a module representative for the survival analysis. As shown in Table 1, turquoise, green, and red modules had a significant association with OS endpoint in univariate analysis. Moreover, significant modules (MEturquoise, MEgreen, and MEred) were selected as the multivariate analysis covariates. We evaluated if the significant modules in combination had a significant effect on survival. As illustrated in Table 1, green and red were significant in multivariate analysis (Supplementary Table S1). That was statistically significant in the log-rank analysis (*P* value = 0.0003).

**Table 1:** A: Univariate survival analysis for gene co-expression modules with overall survival (OS) and progression-free interval (PFI) as endpoints. B: Multivariate Cox regression among Turquoise, Green, and Red modules. A value of 0.05 for the *P* value was defined as the threshold. Significant modules were indicated with the bold *P* value.

Module name	No. genes	OS			PFI			OS		
		HR	*P* value	CI	HR	*P* value	CI	HR	*P* value	CI
		**A: Univariate survival analysis**						**B: Multivariate survival analysis**		
Brown	272	0.8554	0.443	0.57–1.27	0.8886	0.494	0.63–1.25	–	–	–
Turquoise	3747	0.6543	**0.0393**	0.44–0.98	0.698	0.0612	0.45–1.02	0.64	0.069	0.39–1.04
Blue	437	0.815	0.329	0.54–1.23	1.2987	0.133	0.92–1.83	–	–	–
Green	171	0.5979	**0.0125**	0.40–0.89	0.7368	0.0776	0.53–1.03	0.44	**0.0004**	0.28–0.69
Black	55	0.5848	0.467	0.14–2.48	0.7943	0.38	0.47–1.33	–	–	–
Red	99	1.5476	**0.025**	1.06–2.27	1.06891	0.692	0.77–1.49	1.53	**0.059**	0.98–2.39
Yellow	219	0.629	0.604	0.11–3.63	1.1466	0.301	0.89–1.49	–	–	–

Increased expression of genes in green modules indicates a good prognosis related to OS in NULMS (HR = 0.6, 95% CI = 0.4–0.89, *P* = 0.0125); while red module genes shows poor prognosis (HR = 1.55, 95% CI = 1.06–2.27, *P* = 0.025; Table1). For seven modules, survival curves were plotted through Kaplan–Meier. Plots for green and red modules were illustrated (Supplementary Figure S3). Likewise, univariate analysis revealed that 39% and 20% of genes were significant in green and red modules, respectively (*P* value ≤ 0.01) (Supplementary Table S2 and S3). To validate the result of survival analysis, the GSE71119^19^ was used as an independent cohort. Regarding univariate analysis in green and red modules, fifteen genes with the lowest P.Cox value were selected. We ran multivariate Cox regression on selected genes. In the green module, ICAM3, IL18RAP, LCK, CTSW, and GRAP2 were significant. Also, PYCR1, B3GALT6, GALNT1, UNC5B, MEX3A, and DCN were significant in the red module (Supplementary Table S4).

### Identification of hub genes for prognostic modules

We ranked and picked the top 20 genes based on module membership (MM) and intramodular connectivity separately in each module. In the green and red module, 19 out of 20 and 16 out of 20 genes were common in both lists, respectively (Table 2)^20^. Our findings clearly showed that there is a strong positive correlation between MM and intramodular connectivity. Although all the hub genes were significant with OS (*P* < 0.05) in the green module, they were not the most significant genes or one with the least HR related to the OS. The most important hub gene in the green module was in rank 14 in the list (Table 2).

**Table 2:** Hub genes in green and red modules. P.Cox rank and HR rank are the order of genes; based on *P* value and hazard ratio resulted from univariate Cox regression in the modules.

Green module					Red module				
Genes	MM	KWithin	P.Cox rank	HR rank	Genes	MM	kWithin	P.Cox rank	HR rank
LCK	0.99	46.52	14	22	FSCN1	0.91	8.57	10	9
CD3G	0.98	43.27	79	84	PGD	0.88	7.39	32	49
CD2	0.97	42.31	28	56	TPM3	0.86	6.87	59	58
CD3D	0.96	39.09	49	72	PYCR1	0.89	6.72	1	1
SLAMF6	0.96	38.27	47	51	TUBB3	0.89	6.67	47	48
SLA2	0.95	37.98	60	61	COL1A1	0.85	6.44	19	18
SH2D1A	0.95	37.83	48	39	TTYH3	0.88	6.27	70	69
PYHIN1	0.95	36.87	69	63	ULBP2	0.85	6.24	12	14
ZAP70	0.96	36.36	59	66	SEC61A1	0.86	5.77	60	60
CD96	0.95	36.20	36	19	DCBLD1	0.84	5.67	61	66
ITGAL	0.96	35.83	34	62	COL5A1	0.85	5.63	23	23
CD8A	0.94	35.19	31	40	GOLM1	0.85	5.41	48	55
CXCR3	0.95	34.69	25	53	CDH11	0.85	5.39	37	32
CD5	0.95	34.41	41	86	LRRC8D	0.84	5.31	28	25
CD3E	0.96	33.67	50	71	HOXA1	0.85	5.23	68	74
IL2RG	0.95	33.44	35	79	SRPX2	0.87	5.05	6	15
CD247	0.96	32.88	32	34					
UBASH3A	0.94	32.36	77	68					
RLTPR	0.94	32.14	101	104					

In the red module, 13 out of 16 hub genes (81%) had a significant relationship with OS (*P* < 0.05). Except for PYCR1 in the first rank, the hub genes were not the most significant genes or one with the high HR related to the OS.

### Functional enrichment analysis of prognostic module genes

Functional analysis was performed through the DAVID bioinformatics tool for all genes with a *P* value smaller than 0.01 in the green and red modules (Supplementary Table S2 and S3). As shown in Table 3, the green module genes were significantly enriched for immune response, inflammatory response, positive regulation of natural killer cell-mediated cytotoxicity, T cell activation, and B cell activation. Cytokine–cytokine receptor interaction and immunoregulatory interactions between a lymphoid and a non-lymphoid cell were significant pathways.

**Table 3:** Functional annotation terms in the green and red module.

Green module					
Functional annotation term	Count	FDR	Functional annotation term	Count	FDR
**GO-biological process (BP)**			**KEGG pathway**		
Regulation of immune response	14	1.26E−10	Cytokine-cytokine receptor interaction	13	4.61E−06
T cell activation	10	1.37E−10	Primary immunodeficiency	6	0.001058
Immune response	16	4.29E−08	T cell receptor signaling pathway	8	0.001114
**GO-molecular function (MF)**			Natural killer cell-mediated cytotoxicity	7	0.053052
Transmembrane signaling receptor activity	8	0.0117	**REACTOME pathway**		
SH3/SH2 adaptor activity	5	0.0520	Immunoregulatory interactions between a Lymphoid and a non-Lymphoid cell	12	2.68E−07
**GO**−**cellular component**			Generation of second messenger molecules	7	8.55E−−06
T cell receptor complex	7	3.13E−08	Chemokine receptors bind chemokines	7	1.84E−04
External side of plasma membrane	11	4.48E−06	Translocation of ZAP-70 to Immunological synapse	5	0.002181

**Red module**					
Functional annotation term	Count	FDR	Functional annotation term	Count	FDR
**GO-biological process (BP)**			Endoplasmic reticulum lumen	7	0.009425
Collagen fibril organization	7	7.81E−06	Extracellular matrix	7	0.058221
Collagen catabolic process	6	0.002068	**REACTOME pathway**		
Extracellular matrix organization	7	0.033869	Collagen biosynthesis and modifying enzymes	6	0.001231
Collagen biosynthetic process	3	0.033869	ECM proteoglycans	6	0.001231
**GO-molecular function (MF)**			Extracellular matrix organization	4	0.001538
Extracellular exosome	28	0.005448	Collagen degradation	5	0.005712
Extracellular space	17	0.009425			

## Discussion

In this study, we used the WGCNA framework to analyze the mRNA expression data to find essential modules and genes related to clinical information, especially the survival of the NULMS. The studies on this type of cancer are limited, mainly based on network analysis. WGCNA, as an unsupervised algorithm, can establish and detect the relationship between gene expression and clinical traits. In the present study, seven distinct co-expression modules were identified from 5000 most connected genes; two of them were significantly related to OS status in multivariate Cox regression analysis. For more insight and finding biological mechanisms, hub genes were explored. Increased expression of genes in the green module indicated favorable prognosis related to OS in NULMS; while the red module showed poor prognosis associated with OS. Based on univariate Cox regression, the green module's top five most significant genes were ICAM3, NCR3, KLRB1, IL18RAP, and CECR1. In order to GO analysis, most of the genes of the green module were in the plasma membrane (GO:0005886), integral component of membrane (GO:0016021), T cell receptor complex (GO:0042101), immunological synapse (GO:0001772), and alpha–beta T cell receptor complex (GO:0042105). Based on GO biological function, there were enriched in regulation of the immune response (GO:0050776, GO:0006955), T cell activation (GO:0042110), adaptive immune response (GO:0002250), T cell costimulation (GO:0031295), chemokine-mediated signaling pathway (GO:0070098), inflammatory response (GO:0006954), positive regulation of natural killer cell-mediated cytotoxicity (GO:0045954), B cell activation (GO:0042113), and many other critical biological responses which are listed in supplementary files.

The green module deduced that our WGCNA model successfully separated gene expression of immune cells in the tumor microenvironment from cancer cells and other cancer tissues' cellular components. Numerous studies showed the link between immune cell infiltration in the tumor site and better response to therapy and prognosis in carcinomas^21^. For example, infiltration of CD8 + and CD57 + cells (as markers of CD8 + T-cells and NK-cells) in tumors was shown as an independent prognostic factor for a more prolonged disease-free survival^22^. Several studies^23,24^ in cancer favor immune cell infiltration and better survival even in different sarcoma^24^ and Ewing sarcoma^25^. But, there are still some controversies in carcinomas and sarcomas^23,26^.

The immune infiltration may be prominent in response to immunotherapy drugs. Recently, a clinical trial in undifferentiated pleomorphic sarcoma (UPS) revealed a positive correlation between immune infiltration and response to pembrolizumab. Increased percentage of tumor-associated macrophages (TAM); expressing PD-L1 and higher accumulation of activated T cells (CD8 + CD3 + PD-1 +) were associated with better response to Pembrolizumab^27^. Immunotherapy is rapidly developing, and predicting the response to it is an enormous prerequisite. Moreover, finding a suitable target for immunotherapy is of utmost importance^28^. For patients with proper expression of immune markers, available drugs may be applied or a new medication might be designed. For patients or cancer types with lower expression of immune genes, an alternate therapy except for immunotherapy may be useful. This manuscript suggests that NULMS might be a good candidate for immunotherapy.

Based on GO analysis, most of the genes were in extracellular space and the extracellular exosome in the red module. Based on GO biological function, there were enriched for an extracellular matrix, collagen fibril, collagen catabolic process, etc. Genes in this module were enriched in biological processes, including angiogenesis, cell–cell adhesion, protein glycosylation, and protein transport functions (Supplementary Table S3).

The most significant gene in the red module was Pyrroline-5-carboxylate reductase 1 (PYCR1). It is a crucial proline biosynthesis enzyme. Most of the studies showed that this gene is an unfavorable prognostic marker in cancers^29–31^. It is also essential in cell proliferation in NSCLC^32^.

Spermidine synthase (SRM) is an unfavorable tumor marker that is expressed in renal and liver cancer based on Human Protein Atlas. Its function is a polyamine metabolic process based on GO cellular function. It was also showed that inhibition of SRM could slow B cell lymphoma onset in transgenic mice^33^. Studies on this protein are limited, and it is recommended to perform similar investigations for NULMS. Moreover, the SRM inhibitors would be a research line for therapy in this subtype of cancer.

Beta-1, 3-Galactosyltransferase 6 (B3GALT6) was another gene in the red module with a hazard ratio of 2. Based on GO biological function, this protein is vital in glycosaminoglycan synthesis and protein glycosylation. Mutation of this gene was also reported in connective tissue disorder^34^. Few studies have been performed on this protein in the cancer area. But, protein glycosylation was studied well in cancer formation, microenvironment, and metastasis^35^. Studies on this protein may contribute to our better understanding of NULMS.

According to Human Protein Atlas, high expression of NECAP2 is a favorable and unfavorable prognostic factor in colorectal and liver cancer, respectively. It was shown that NECAP2 is a crucial factor for recruiting AP-1 to early endosomes and the efficient recycling of EGFR. It controls the clathrin coat recruitment of endosomes for the recycling of EGFR^36^. EGFR signaling is one of the important pathways in cancer. Dysregulated intracellular trafficking of the EGFR family of receptor tyrosine kinases plays a critical role in oncogenesis^37^.

Moreover, metastasis is caused by increased cancer cell migration and invasion and is the leading cause of cancer-related mortality. NECAP2 function is also essential for the fast recycling of integrin αvβ3 and integrin αvβ3-dependent migration and cancer cell invasion^38^. The result of the present study showed that NECAP2 is a marker for poor prognosis. Thus, therapies on controlling endosome trafficking may be useful in NULMS.

Myod inhibitor (MDFI) is a tumor suppressor gene and can inhibit proliferation in breast cancer 4T1 cell line^39^. Down-regulation of MDFI through hyper-methylation may be a risk of NSCLC in young, smoker women^40^. The study of this protein in cancer is also limited and could be continued in LMS. It is noteworthy that validation of significant genes in both modules confirmed that these genes were important in an independent dataset and could be proper candidates for further experimental and clinical analysis.

Hub gene analysis showed that although all hub genes were significant with OS in the green and red modules, they were not the most significant genes or one with the least HR related to the OS, except for PYCR1. The effect of hub genes in survival was investigated in many studies and hub genes were introduced as important prognostic markers^14,41^. It is important to note that we should look at relapse as a consequence of complex mechanisms, and nodes, hub genes, are not the best options for predicting them. Every single gene in a significant module may have a cumulative effect on survival, and pinpointing nodes can not be the whole story.

## Materials and methods

**Figure 5:**
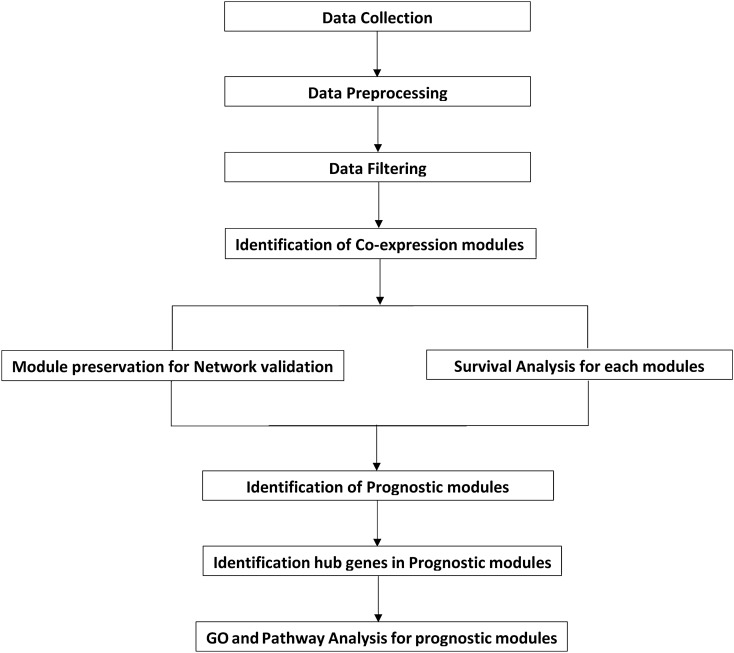
Flow chart of sequential steps for data preprocessing and analysis. Data Prep-aration includes data collection, preprocessing, and filtering. Three data sets were downloaded; the first one was TCGA NULMS. The GSE45510 was used for module preservation, and the GSE71119 was applied for validation of sur-vival analysis. The next step is NULMS co–expression network construction was based on the prepared data. Then, constructed modules were validated through the module preservation process and survival analysis was performed on validated modules. Identification of prognostic modules was performed in the next step. Then, hub genes in prognostic modules were investigated. Final-ly, the biological processes and different pathways; related to identified mod-ules were analyzed.

The research design and all steps of this study are presented in the flowchart of Fig. 5. Data collection, preprocessing, and filtering were executed in three steps that were performed before constructing the co-expression network. NULMS co-expression network was constructed on preprocessed data, and the validation process was achieved through the module preservation. Moreover, survival analysis was performed on preserved modules. The identification of prognostic modules was the next step of this study. Subsequently, hub genes in prognostic modules were investigated. Finally, the biological process and different pathways; related to identified modules were analyzed. R platform (version 3.6.1) was used for the computational analysis.

### Dataset and preprocessing

Gene expression and clinical data of two datasets were used. The first one, as the main dataset, was from TCGA and included 74 NULMS cases. Those cases' clinical information was also obtained from the supplementary section of a TCGA paper^42^ on the integrated TCGA pan-cancer clinical data resource. In connection with biological data, The Cancer Genome Atlas (TCGA) was utilized as the primary source of RNA-seq. TCGA is a project, including different omics data related to various cancers. Through that project, more than 20,000 cancer and normal samples were collected (https://www.cancer.gov/tcga).

The second dataset (GSE45510) was downloaded from NCBI Gene Expression Omnibus, which included 50 NULMS cases^2^, and it is used as the validation dataset.

HTSeq–Count files of TCGA NULMS were downloaded through the "TCGAbiolinks" package^43^. TCGA HTseq-counts were normalized based on the Transcripts Per Million (TPM) method. These data were transferred to a new space; using the log2 function. "BatchQC" package^44^ was used for finding batch effects in the TCGA dataset. Batch effects correction was done; using the "sva"package^45^. For this purpose, the ComBat function was used with the parametric adjustment (Supplementary Figure S1). Among NULMS cases in TCGA, two patients "TCGA_IE_A3OV", "TCGA_K1_A6RT" were removed because they belonged to batches with just one patient. Hierarchical clustering was performed through samples for finding outliers. Also, the Adaptive branch pruning of hierarchical clustering (dendrogram) was applied by the "dynamicTreeCut" package (Supplementary Figure S2). Through that process, "TCGA_DX_A3UB" was detected as an outlier. Eventually, in the last step of preprocessing, both datasets were checked for missing entries and zero-variance genes; using the goodSamplesGenes function in the WGCNA package.

In this study, the expression matrix was constructed; using protein-coding genes. The analysis was restricted to the most connected genes with non-zero variance. At last, 5000, most connected genes were chosen by applying the softConnectivity function from the WGCNA package^15^, and connectivity was calculated between genes.

### Weighted gene co-expression network construction

The co-expression network was constructed based on WGCNA functions. In this study, biweight midcorrelation (bicor) was used to compute the correlation between each pair of genes because of its robustness to noise in comparison to the Pearson correlation coefficient. Between 3 types of co-expression networks, we used the signed network. In this network, zero correlation gives rise to a non-zero adjacency, and the similarity is defined as (1 + cor)/2.

The correlation matrix was transferred to the adjacency matrix through the adjacency function from the WGCNA package with power β.

That power was calculated; using the pickSoftThreshold function. So, we used the arguments (corFnc = "bicor", corOptions = list (maxPOutliers = 0.1), network type = “signed”, power = “β”) to meet the need of scale-free topology property of the co-expression network. A generalized version of Topological Overlap Measure (TOM) was utilized to find clusters of highly co-expressed genes (modules).TOM in the TOMsimilarity function was applied, converting similarity values for each pair of genes to the new matrix, which was non-negative and symmetric. TOM calculates the similarities based on the number of shared neighbors between gene pairs in the resulting co-expression network^46^. Since TOM-based dissimilarity has better performance for the distinction gene module, in WGCNA, 1-TOM was used instead of TOM^47^. Hierarchical clustering was built by the average linkage hierarchical clustering algorithm implemented in the hClust function. Gene modules, groups of genes with a similar expression, were identified with the cutreeDynamic function^48^. In this function, the “deepSplit” argument value was 2, and a minimum cluster size was 50.

The module eigengene (ME) is a robust and proper representative for each module. It is the first principle component in each module that covers the highest percentage of variance for expression values of all genes in a module^15^. The moduleEigengenes function calculated the MEs. The close modules were merged through the mergeCloseModules function and determining of MEs threshold was applied on hierarchical clustering of computed modules eigengene.

### Validation of identified co-expressed modules

If a module in the reference dataset is not determined randomly, it will be reproduced in other independent datasets across different conditions. In this study, validation of co-expressed modules in the TCGA NULMS dataset was done by an independent dataset (GSE45510); explained in the dataset and preprocessing part. Module preservation statistics was used to validate whether a defined module in one data set could also be found in another data set. The WGCNA used two composite preservation statistics for module preservation: First, Z-summary distinguished preserved modules from non-preserved ones through the permutation test (nPermutations = 200). Median rank is another statistic to compare the amount of preservation among modules. Compare the two modules, the one with a higher median rank was considered to have a lower preservation tendency^49^.

### Finding modules of interest

#### Module-trait relationship

The relationship between modules and traits was calculated by ME. In other words, we applied ME for calculating the Pearson correlation coefficient between each module and traits through cor() function. Clinical traits included age, OS, DSS, DFI, and PFI status/time. Among the different survival endpoints, OS and PFI were selected for survival analysis due to complete available clinical data and no missing values.

#### Survival analysis

Survival^50^, Survminer^51^, and RegParallel^52^ packages were used to identify the module-survival relationship. The ME, as the representative of each module, was selected to define the association of each module with OS and PFI. Therefore, for multigene associations, each ME was dichotomized into positive and negative values^53^. Then, univariate Cox regression, the hazard ratio (HR), and K-M plot were applied for each module; using log-rank tests. In the next step, modules with *P* value ≤ 0.05 in univariate were selected for multivariate Cox regression. Finally, single-gene survival analysis was done on genes in significant prognostic modules.

### Identification of hub genes

A hub gene is a highly-interconnected node in a module with the highest intra-modular connectivity that defines as module membership (MM)^20^. Hub genes were identified by calculating gene connectivity; using the intramodularConnectivity function from the WGCNA in the whole network (kTotal) and each module (kWithin). The MM, which is also a measure in WGCNA, assesses the correlation between a gene and the ME in a module. In this study, two lists of genes with the highest connectivity and MM were selected. In the end, hub genes were chosen through the intersection of these two lists.

### Functional annotation

Gene enrichment analysis was performed for the genes within the significant modules; using the Database for Annotation, Visualization, and Integrated Discovery (DAVID). Depending on DAVID outcome, gene ontology and various pathways for selected genes were investigated. In the pathway analysis, we investigated the Kyoto Encyclopedia of Genes and Genomes (KEGG), Reactome, and Biological Biochemical Image Database (BBID). *P* value ≤ 0.05 and false discovery rate (FDR) smaller than 0.05 were considered as the cut-off for determining significant terms.

## Conclusion

In summary, WGCNA was used to construct a gene co-expression network. ICAM3, NCR3, KLRB1, IL18RAP, and CECR1 were identified as good prognosis genes, most of them related to immune cells. Our results revealed the immune cell infiltration as a favorable prognostic factor. Moreover, PYCR1, SRM, and MDFI negatively affected the OS. These genes are related to angiogenesis, cell–cell adhesion, protein glycosylation, and protein transport functions. We also found hub genes the most significant of which were LCK, FSCN1, CD3G, PGD, CD2, and TPM3. Our findings confirmed prior investigations that the hub genes were not necessarily the most effective genes related to the OS. The genes found in this study were validated in an independent cohort and provided a virtuous gene list for further experimental analysis. Experiments investigating the mechanism of function of these genes and multi-omics data integration in NULMS are further warranted.

## Supplementary Information

Supplementary Information 1.

Supplementary Information 2.

Supplementary Information 3.

Supplementary Information 4.

Supplementary Information 5.

Supplementary Information 6.

Supplementary Information 7.
