# Supplementary material for: Gene co-expression network analysis reveals immune cell infiltration as a favorable prognostic marker in non-uterine leiomyosarcoma
Source: Sci Rep. 2021 Jan 27;11:2339. doi: 10.1038/s41598-021-81952-8 (PMC7840729; doi:10.1038/s41598-021-81952-8)

**Gene Co-Expression Network Analysis Reveals Immune Cell Infiltration as a Favorable Prognostic Marker in Non-Uterine Leiomyosarcoma**

Mohammad Darzi ^1^, Saeid Gorgin ^1*^, Keivan Majidzadeh-A ^2^ & Rezvan Esmaeili ^2*^

^1^ Department of Electrical Engineering and Information Technology, Iranian Research Organization for Science and Technology (IROST), Tehran, Iran; modarzi@irost.ir , gorgin@irost.ir

^2^ Genetics Department, Breast Cancer Research Center, Motamed Cancer Institute, ACECR, Tehran, Iran; kmajidzadeh@acecr.ac.ir , esmaeili.rezvan@gmail.com

* Correspondence: esmaeili.rezvan@gmail.com, gorgin@irost.ir (Tel: (+98) 9125191902, (+9821) 56276020)

Suplementary Figure S3: The Kaplan–Meier plot. The left plot belongs to green modules, and the right one is for the red.

K-M plot for red module

K-M plot for green module


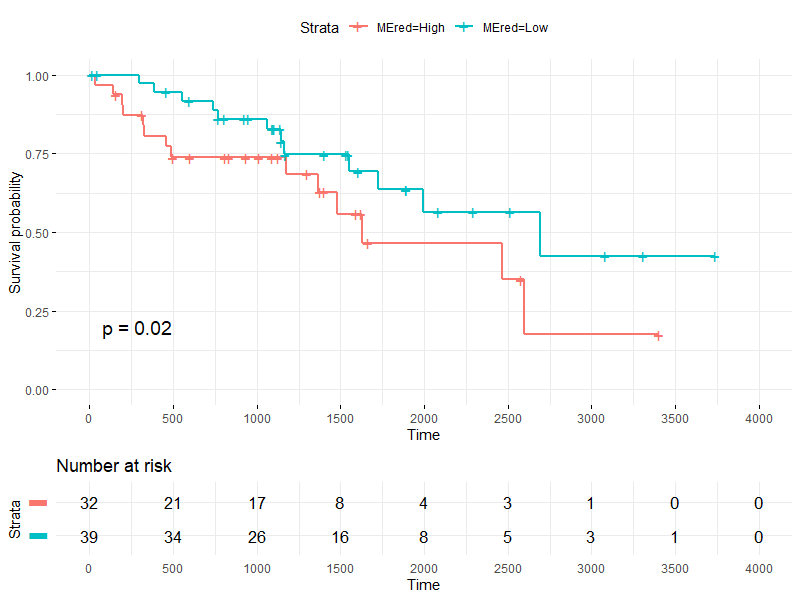

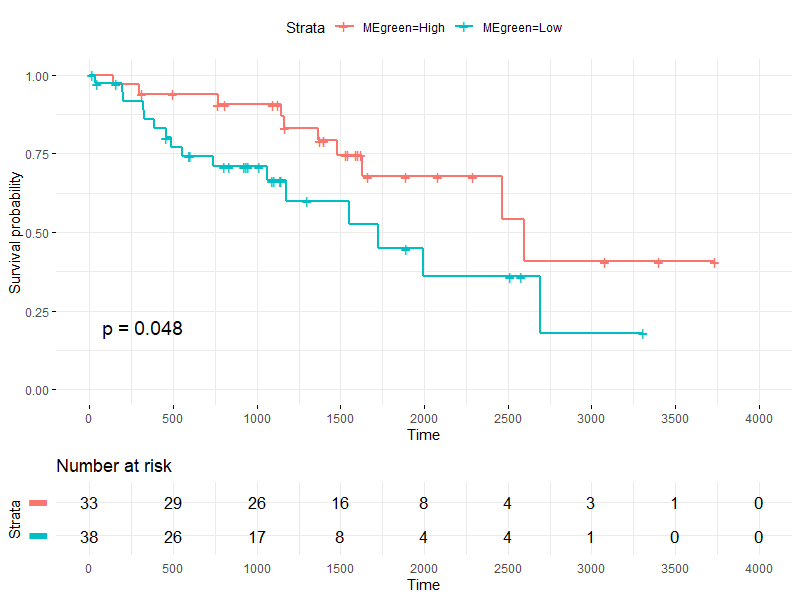

Supplement: Supplementary file 3 — Supplementary Information 3. [file 41598_2021_81952_MOESM3_ESM.docx]
