# Supplementary material for: Gene co-expression network analysis reveals immune cell infiltration as a favorable prognostic marker in non-uterine leiomyosarcoma
Source: Sci Rep. 2021 Jan 27;11:2339. doi: 10.1038/s41598-021-81952-8 (PMC7840729; doi:10.1038/s41598-021-81952-8)
Supplement: Supplementary file 4 — Supplementary Information 4. [file 41598_2021_81952_MOESM4_ESM.docx]

**Gene Co-Expression Network Analysis Reveals Immune Cell Infiltration as a Favorable Prognostic Marker in Non-Uterine Leiomyosarcoma**

Mohammad Darzi ^1^, Saeid Gorgin ^1*^, Keivan Majidzadeh-A ^2^ & Rezvan Esmaeili ^2*^

^1^ Department of Electrical Engineering and Information Technology, Iranian Research Organization for Science and Technology (IROST), Tehran, Iran; modarzi@irost.ir , gorgin@irost.ir

^2^ Genetics Department, Breast Cancer Research Center, Motamed Cancer Institute, ACECR, Tehran, Iran; kmajidzadeh@acecr.ac.ir , esmaeili.rezvan@gmail.com

* Correspondence: esmaeili.rezvan@gmail.com, gorgin@irost.ir (Tel: (+98) 9125191902, (+9821) 56276020)

Suplementary Table S1: Multivariate Cox regression results in R console for three Modules include turquoise, green, and red.

n= 71, number of events= 26

coef exp(coef) se(coef) z Pr(>|z|)

MEturquoise -0.4493 0.6380 0.2471 -1.818 0.069047 .

MEred 0.4262 1.5314 0.2264 1.882 0.059823 .

MEgreen -0.8232 0.4390 0.2326 -3.539 0.000402 ***

---

Signif. codes: 0 ‘***’ 0.001 ‘**’ 0.01 ‘*’ 0.05 ‘.’ 0.1 ‘ ’ 1

exp(coef) exp(-coef) lower .95 upper .95

MEturquoise 0.638 1.567 0.3931 1.0357

MEred 1.531 0.653 0.9825 2.3869

MEgreen 0.439 2.278 0.2783 0.6926

Concordance= 0.711 (se = 0.07 )

Likelihood ratio test= 19.43 on 3 df, p=2e-04

Wald test = 19.11 on 3 df, p=3e-04

Score (logrank) test = 19.07 on 3 df, p=3e-04

The result summarized in table 1:

**Table1.**Multivariate cox regression among three modules

|  | OS | | |
| --- | --- | --- | --- |
|  | HR | P-value | CI |
| MEturquoise | 0.64 | 0.069 | 0.39-1.04 |
| MEgreen | 0.44 | 0.0004 | 0.28-0.69 |
| MEred | 1.53 | 0.059 | 0.98-2.39 |

MEs values for each module relate to each sample.

| Module eigengene  Sample IDs | MEblue | MEturquoise | MEblack | MEyellow | MEred | MEbrown | MEgreen |
| --- | --- | --- | --- | --- | --- | --- | --- |
| TCGA_3B_A9HP | -0.19589 | -0.09827 | -0.03918 | -0.03202 | 0.081025 | 0.104316 | 0.035172 |
| TCGA_3B_A9HQ | -0.01671 | -0.00875 | -0.05304 | -0.0341 | -0.09037 | 0.145581 | 0.214204 |
| TCGA_3B_A9HR | 0.10276 | 0.133223 | -0.02179 | -0.03937 | -0.13904 | -0.2089 | -0.17955 |
| TCGA_3B_A9HS | -0.10984 | -0.16168 | -0.01743 | -0.00118 | 0.173488 | 0.121521 | 0.161293 |
| TCGA_3B_A9HT | -0.02877 | -0.17948 | -0.01743 | -0.01469 | 0.151192 | 0.249558 | 0.26735 |
| TCGA_3B_A9HU | 0.034637 | 0.006567 | -0.01743 | -0.01088 | -0.09679 | 0.141972 | 0.21815 |
| TCGA_3B_A9HX | -0.05881 | -0.03821 | -0.01743 | -0.01395 | 0.157889 | 0.000154 | -0.00954 |
| TCGA_3B_A9HY | 0.205056 | 0.219144 | -0.01743 | -0.01907 | 0.017638 | -0.02852 | 0.002428 |
| TCGA_3B_A9HZ | -0.1396 | 0.014108 | -0.01743 | -0.01582 | -0.03854 | 0.09584 | 0.06498 |
| TCGA_3B_A9I0 | -0.06509 | -0.06445 | -0.01743 | -0.01961 | -0.15173 | 0.075467 | 0.05507 |
| TCGA_3B_A9I3 | 0.041589 | -0.03125 | -0.01743 | -0.00588 | 0.05001 | -0.04141 | 0.008015 |
| TCGA_DX_A3U7 | 0.238266 | 0.104513 | -0.01025 | -0.0111 | -0.00472 | -0.12221 | -0.07972 |
| TCGA_DX_A3U8 | -0.11709 | -0.08432 | -0.01025 | -0.00291 | 0.131231 | 0.302826 | 0.202524 |
| TCGA_DX_A3U9 | 0.17077 | 0.033457 | -0.01746 | -0.00474 | 0.021786 | -0.15665 | -0.12178 |
| TCGA_DX_A3UA | -0.04406 | -0.04122 | -0.01746 | -0.01898 | 0.257475 | 0.079409 | -0.02575 |
| TCGA_DX_A3UC | 0.155382 | 0.065111 | -0.01746 | -0.01815 | -0.08195 | 0.035379 | 0.063919 |
| TCGA_DX_A3UD | 0.069777 | -0.05878 | -0.01746 | -0.01062 | -0.15264 | -0.05346 | 0.017818 |
| TCGA_DX_A3UE | -0.04024 | -0.02046 | -0.01746 | -0.01428 | -0.09408 | -0.13557 | -0.14468 |
| TCGA_DX_A3UF | 0.060732 | 0.006083 | -0.01746 | -0.0138 | 0.017672 | 0.060923 | 0.068933 |
| TCGA_DX_A48J | -0.18472 | 0.128336 | -0.01746 | -0.00566 | -0.1211 | -0.08269 | 0.002859 |
| TCGA_DX_A48K | -0.04682 | -0.09266 | -0.01746 | -0.01864 | 0.257809 | 0.19189 | 0.073639 |
| TCGA_DX_A48L | 0.078195 | 0.116727 | -0.01746 | -0.00698 | -0.03227 | -0.13604 | -0.12163 |
| TCGA_DX_A48O | -0.06319 | 0.009954 | -0.01746 | -0.00505 | -0.11662 | -0.0334 | 0.05901 |
| TCGA_DX_A48P | -0.12718 | -0.02828 | -0.01746 | -0.0184 | -0.08027 | 0.122923 | 0.086555 |
| TCGA_DX_A48R | 0.144519 | 0.097801 | -0.01746 | -0.01061 | -0.14423 | -0.21632 | -0.16639 |
| TCGA_DX_A48U | -0.14474 | 0.048333 | -0.01746 | -0.01848 | -0.16009 | -0.14488 | -0.10173 |
| TCGA_DX_A6B7 | -0.08944 | 0.02477 | -0.01746 | -0.01505 | -0.15923 | -0.15751 | -0.11235 |
| TCGA_DX_A6B8 | 0.041823 | -0.17189 | -0.01746 | -0.0189 | 0.291289 | -0.03447 | -0.15713 |
| TCGA_DX_A6B9 | -0.12801 | 0.107119 | -0.02585 | 0.036226 | -0.02931 | -0.04466 | -0.00298 |
| TCGA_DX_A6BA | 0.231818 | 0.125132 | -0.01746 | -0.01922 | -0.01767 | 0.026688 | -0.04312 |
| TCGA_DX_A6BB | -0.07915 | 0.044689 | -0.02467 | 0.02416 | -0.15615 | -0.14336 | -0.08874 |
| TCGA_DX_A6Z2 | -0.00069 | 0.065717 | -0.03448 | -0.00842 | -0.04864 | 0.125717 | 0.187151 |
| TCGA_DX_A7EL | -0.09247 | 0.016836 | -0.03551 | -0.02945 | -0.11392 | 0.080481 | 0.219279 |
| TCGA_DX_A7EM | 0.195718 | 0.175511 | -0.02096 | -0.02429 | -0.03754 | 0.008475 | 0.026853 |
| TCGA_DX_A7EN | 0.222419 | 0.086229 | -0.01743 | 0.01059 | -0.15444 | -0.22654 | -0.18684 |
| TCGA_DX_A8BZ | -0.26836 | -0.39411 | -0.01574 | -0.00961 | 0.044585 | 0.089284 | -0.11349 |
| TCGA_FX_A3NJ | -0.01734 | 0.135522 | -0.01967 | -0.02511 | -0.10749 | -0.07041 | -0.0495 |
| TCGA_HB_A5W3 | -0.06338 | 0.125372 | -0.01025 | -0.00382 | -0.07197 | -0.00915 | -0.0317 |
| TCGA_HS_A5N7 | 0.034793 | -0.10822 | -0.02798 | -0.0379 | 0.051472 | 0.096063 | -0.01356 |
| TCGA_IE_A4EI | 0.054229 | 0.066452 | -0.05316 | -0.0303 | -0.0405 | -0.0651 | -0.12987 |
| TCGA_IE_A4EK | -0.17323 | 0.073853 | -0.05316 | -0.05176 | -0.15579 | -0.22719 | -0.1454 |
| TCGA_IF_A4AJ | 0.061071 | 0.005525 | -0.05316 | -0.0167 | -0.00769 | 0.132101 | 0.173764 |
| TCGA_IF_A4AK | 0.200217 | 0.155084 | -0.05316 | -0.03678 | 0.018856 | -0.0263 | 0.053225 |
| TCGA_JV_A5VE | 0.097803 | 0.040129 | -0.01025 | 0.018608 | 0.076094 | 0.075088 | 0.137935 |
| TCGA_JV_A5VF | -0.0012 | -0.23978 | -0.01025 | -0.00131 | 0.179889 | -0.0607 | -0.15961 |
| TCGA_K1_A3PO | -0.20081 | -0.14875 | -0.01385 | -0.01682 | 0.090252 | 0.110234 | 0.109219 |
| TCGA_K1_A42W | -0.00401 | -0.14032 | -0.05316 | -0.05205 | -0.07178 | 0.065812 | -0.04289 |
| TCGA_K1_A6RV | -0.0106 | -0.19279 | 0.006599 | 0.029871 | 0.136346 | 0.065101 | -0.07073 |
| TCGA_MB_A5YA | -0.10397 | -0.10382 | -0.01025 | -0.01064 | -0.16932 | -0.05751 | -0.07034 |
| TCGA_MB_A8JL | -0.10054 | -0.01597 | -0.03551 | -0.02883 | 0.232911 | -0.08949 | -0.11926 |
| TCGA_MJ_A68H | 0.072636 | 0.100587 | -0.01746 | -0.01815 | -0.02775 | -0.06246 | -0.06353 |
| TCGA_MO_A47R | -0.0669 | -0.00699 | -0.05342 | -0.04082 | 0.096288 | -0.04308 | -0.09152 |
| TCGA_PC_A5DK | -0.05762 | 0.146319 | -0.00737 | -0.02664 | -0.13853 | 0.06855 | 0.191514 |
| TCGA_PC_A5DL | -0.08501 | 0.078412 | -0.01729 | -0.03467 | -0.10477 | 0.002276 | 0.068285 |
| TCGA_PC_A5DM | 0.040547 | -0.16861 | -0.03113 | -0.03173 | 0.097614 | -0.06187 | -0.13884 |
| TCGA_PC_A5DN | -0.10093 | -0.0175 | -0.0319 | -0.02827 | 0.105496 | 0.020179 | -0.02222 |
| TCGA_PC_A5DO | 0.032004 | 0.088261 | -0.03638 | 0.02265 | 0.024648 | 0.052716 | 0.090192 |
| TCGA_PC_A5DP | 0.013251 | 0.044364 | -0.04285 | -0.02956 | -0.09534 | -0.2299 | -0.1685 |
| TCGA_QQ_A5VB | -0.0445 | -0.27112 | 0.020608 | -0.03018 | 0.178493 | 0.170281 | 0.090475 |
| TCGA_QQ_A5VC | 0.031012 | -0.11634 | 0.813916 | 0.031273 | 0.117128 | -0.0569 | -0.05439 |
| TCGA_QQ_A5VD | -0.00078 | -0.04454 | 0.452126 | 0.049877 | 0.067415 | 0.174702 | 0.152994 |
| TCGA_QQ_A8VF | 0.036998 | -0.05307 | 0.007396 | 0.037549 | 0.171783 | 0.035768 | 0.18546 |
| TCGA_WK_A8XS | 0.133423 | 0.115728 | -0.01227 | -0.00643 | 0.045155 | -0.1058 | -0.0735 |
| TCGA_WK_A8XX | 0.033827 | 0.038422 | -0.01574 | -0.00661 | -0.02466 | 0.123945 | 0.023291 |
| TCGA_WK_A8XY | 0.100618 | 0.110412 | -0.01574 | -0.01551 | -0.07071 | -0.14247 | -0.14128 |
| TCGA_WK_A8XZ | 0.084612 | 0.149579 | -0.01574 | -0.01014 | -0.06817 | -0.03068 | -0.01173 |
| TCGA_X6_A7WA | -0.05917 | 0.088062 | 0.004833 | -0.029 | -0.07423 | 0.053237 | -0.01189 |
| TCGA_X6_A7WC | -0.12267 | -0.22635 | 0.282227 | 0.977939 | 0.151075 | 0.117869 | 0.147297 |
| TCGA_X6_A7WD | 0.179559 | 0.105371 | -0.07383 | -0.04579 | 0.030711 | -0.09643 | -0.14215 |
| TCGA_X6_A8C5 | -0.18725 | -0.03143 | -0.03551 | -0.01732 | -0.10039 | 0.11413 | 0.078865 |
| TCGA_X9_A971 | 0.240745 | 0.062609 | -0.04929 | -0.04598 | 0.025733 | -0.13444 | -0.12984 |
