# Supplementary material for: Gene co-expression network analysis reveals immune cell infiltration as a favorable prognostic marker in non-uterine leiomyosarcoma
Source: Sci Rep. 2021 Jan 27;11:2339. doi: 10.1038/s41598-021-81952-8 (PMC7840729; doi:10.1038/s41598-021-81952-8)
Supplement: Supplementary file 7 — Supplementary Information 7. [file 41598_2021_81952_MOESM7_ESM.docx]

**Gene Co-Expression Network Analysis Reveals Immune Cell Infiltration as a Favorable Prognostic Marker in Non-Uterine Leiomyosarcoma**

Mohammad Darzi ^1^, Saeid Gorgin ^1*^, Keivan Majidzadeh-A ^2^ & Rezvan Esmaeili ^2*^

^1^ Department of Electrical Engineering and Information Technology, Iranian Research Organization for Science and Technology (IROST), Tehran, Iran; modarzi@irost.ir , gorgin@irost.ir

^2^ Genetics Department, Breast Cancer Research Center, Motamed Cancer Institute, ACECR, Tehran, Iran; kmajidzadeh@acecr.ac.ir , esmaeili.rezvan@gmail.com

* Correspondence: esmaeili.rezvan@gmail.com, gorgin@irost.ir (Tel: (+98) 9125191902, (+9821) 56276020)

Supplementary Table S4: Survival Analysis Validation in Green and Red modules through an independent cohort.

The GSE71119 was used as an independent cohort to validate the result of the survival analysis. From red and green modules, fifteen genes with the lowest p-value were picked up. Then, the expression values of selected genes extract from the cohort. The multivariate Cox regression was performed on these genes.

The R output for multivariate Cox analysis on selected genes in the green module. Gene’s p-value with “*” or “**” are significant genes

n= 35, number of events= 18

coef exp(coef) se(coef) z Pr(>|z|)

CCR2 0.46125 1.58606 0.38628 1.194 0.23244

CECR1 0.89436 2.44577 0.74273 1.204 0.22853

CTSW -1.57614 0.20677 0.61467 -2.564 0.01034 *

CXCR6 0.98868 2.68769 1.01241 0.977 0.32879

GRAP2 -1.96053 0.14078 0.77087 -2.543 0.01098 *

ICAM3 1.36700 3.92358 0.71257 1.918 0.05506 .

IL18RAP -2.24902 0.10550 0.74568 -3.016 0.00256 **

KLRB1 -0.35649 0.70013 0.45571 -0.782 0.43406

LCK 3.96865 52.91304 2.01947 1.965 0.04939 *

LTB 0.54762 1.72913 0.63605 0.861 0.38926

LY9 1.07244 2.92252 0.63755 1.682 0.09254 .

NCR3 -0.04814 0.95300 0.21369 -0.225 0.82176

SIT1 -3.40369 0.03325 1.23968 -2.746 0.00604 **

TBX21 0.60805 1.83684 0.77774 0.782 0.43433

TIFAB -0.15075 0.86007 0.16126 -0.935 0.34988

---

Signif. codes: 0 ‘***’ 0.001 ‘**’ 0.01 ‘*’ 0.05 ‘.’ 0.1 ‘ ’ 1

exp(coef) exp(-coef) lower .95 upper .95

CCR2 1.58606 0.6305 0.743910 3.3816

CECR1 2.44577 0.4089 0.570426 10.4866

CTSW 0.20677 4.8363 0.061984 0.6898

CXCR6 2.68769 0.3721 0.369498 19.5500

GRAP2 0.14078 7.1031 0.031073 0.6379

ICAM3 3.92358 0.2549 0.970824 15.8571

IL18RAP 0.10550 9.4784 0.024465 0.4550

KLRB1 0.70013 1.4283 0.286599 1.7103

LCK 52.91304 0.0189 1.010617 2770.3774

LTB 1.72913 0.5783 0.497073 6.0150

LY9 2.92252 0.3422 0.837664 10.1963

NCR3 0.95300 1.0493 0.626896 1.4487

SIT1 0.03325 30.0748 0.002928 0.3776

TBX21 1.83684 0.5444 0.399996 8.4350

TIFAB 0.86007 1.1627 0.627005 1.1798

Concordance= 0.851 (se = 0.051 )

Likelihood ratio test= 24.16 on 15 df, p=0.06

Wald test = 14.09 on 15 df, p=0.5

Score (logrank) test = 17.51 on 15 df, p=0.3

The R output for multivariate Cox analysis on selected genes in the red module. Gene’s p-value with “*” or “**” are significant genes.

n= 35, number of events= 18

coef exp(coef) se(coef) z Pr(>|z|)

PYCR1 1.15840 3.18483 0.40466 2.863 0.00420 **

SRM -0.08356 0.91984 0.69269 -0.121 0.90399

MDFI 0.30016 1.35007 0.22442 1.337 0.18106

B3GALT6 -1.61053 0.19978 0.73223 -2.199 0.02784 *

GALNT1 4.33475 76.30605 1.34834 3.215 0.00131 **

SRPX2 -0.28158 0.75459 0.38960 -0.723 0.46984

UNC5B -1.30534 0.27108 0.48876 -2.671 0.00757 **

MEX3A -1.67657 0.18702 0.60192 -2.785 0.00535 **

MXRA5 -0.37392 0.68803 0.37155 -1.006 0.31422

FSCN1 0.58464 1.79435 0.66335 0.881 0.37813

RIN1 0.75902 2.13618 0.60874 1.247 0.21244

ULBP2 -0.39485 0.67378 0.39960 -0.988 0.32310

MARCKS 0.86419 2.37307 1.15668 0.747 0.45499

DCN -0.65262 0.52068 0.31793 -2.053 0.04010 *

COL5A3 0.23425 1.26397 0.21954 1.067 0.28595

---

Signif. codes: 0 ‘***’ 0.001 ‘**’ 0.01 ‘*’ 0.05 ‘.’ 0.1 ‘ ’ 1

exp(coef) exp(-coef) lower .95 upper .95

PYCR1 3.1848 0.31399 1.44093 7.0393

SRM 0.9198 1.08715 0.23664 3.5755

MDFI 1.3501 0.74070 0.86963 2.0960

B3GALT6 0.1998 5.00544 0.04756 0.8391

GALNT1 76.3060 0.01311 5.43060 1072.1857

SRPX2 0.7546 1.32522 0.35163 1.6193

UNC5B 0.2711 3.68893 0.10401 0.7065

MEX3A 0.1870 5.34716 0.05748 0.6085

MXRA5 0.6880 1.45343 0.33216 1.4252

FSCN1 1.7944 0.55730 0.48895 6.5849

RIN1 2.1362 0.46812 0.64786 7.0437

ULBP2 0.6738 1.48416 0.30788 1.4746

MARCKS 2.3731 0.42139 0.24589 22.9023

DCN 0.5207 1.92056 0.27922 0.9710

COL5A3 1.2640 0.79116 0.82199 1.9436

Concordance= 0.893 (se = 0.042 )

Likelihood ratio test= 35.61 on 15 df, p=0.002

Wald test = 17.23 on 15 df, p=0.3

Score (logrank) test = 29.83 on 15 df, p=0.01
